# Management of a patient with a severely infraoccluded primary molar and hypodontia

**DOI:** 10.1002/ccr3.6482

**Published:** 2022-11-15

**Authors:** Alaina Ng, Desmond Ong, Phillip Goh

**Affiliations:** ^1^ School of Dentistry The University of Queensland Herston Queensland Australia; ^2^ Private Practice – Townsville, School of Dentistry, Discipline of Orthodontics The University of Queensland Herston Queensland Australia; ^3^ Private Practice – Melbourne, School of Dentistry, Discipline of Orthodontics The University of Queensland Herston Queensland Australia

**Keywords:** hypodontia, infraoccluded primary molar, management

## Abstract

Infraocclusion of a primary molar is a relatively common occurrence which seldom leads to serious complications, particularly when the succedaneous permanent tooth is present. Despite the relative infrequency of unfavorable outcomes, an infraoccluded primary molar may be associated with other dental anomalies, including hypodontia. If diagnosis is delayed, infraocclusion of a primary molar can become severe, with associated negative effects on the adjacent teeth and alveolar bone. This emphasizes the need for routine and periodic orthodontic assessment of growing patients. Any deviation from the normal eruption sequence of teeth should be diagnosed and managed in a timely manner, to preserve dental arch integrity and reduce the potential for undesirable outcomes. This case report demonstrates how comprehensive assessment of the dentition, logical treatment planning and careful management of challenging orthodontic tooth movements can combine to provide a pleasing treatment outcome.

## Graphical abstract

Successful mandibular arch space closure following removal of a severely infraoccluded primary molar.

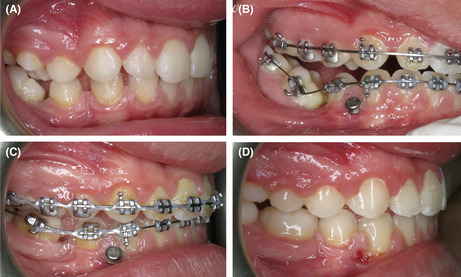

## 1 INTRODUCTION

Infraocclusion is a condition that occurs when the occlusal surface of a tooth is located below the occlusal plane of the adjacent teeth. Submergence is a synonymous term also used to describe this clinical presentation, which most commonly affects primary molar teeth.^1^, ^2^, ^3^ Infraocclusion of primary teeth is caused by ankylosis of the cementum to the surrounding alveolar bone, thus impeding its eruption.^1^, ^2^, ^3^ The incidence of ankylosis is approximately 1.3%–38.5% for primary molars.^1^ Ankylosis can occur at any time and results in the primary tooth failing to keep pace with the normal vertical alveolar bone height changes in a growing individual. Therefore, ankylosis is the phenomenon which results in the lack of tooth eruption and subsequently leads to the clinical presentation of infraocclusion.

The prevalence of this condition is also affected by age.^1^, ^2^, ^3^, ^4^ Infraocclusion of the first primary molar is reported to be more common in children under the age of nine and shows a general increase in incidence after the age of three.^1^, ^2^, ^3^ In comparison, the prevalence of infraoccluded second primary molars increases after the age of five and is more prevalent after the age of nine.^1^, ^2^, ^3^

The prevalence of infraocclusion is significantly higher in the primary dentition compared with the permanent dentition and more commonly found in the mandible as opposed to the maxilla, with no significant gender dimorphism reported. The most commonly affected teeth are the mandibular first and second primary molars, with no predilection between the left and right sides. It has been reported that 1.3%–8.9% of children are affected by infraocclusion of one or more of their primary teeth.^1^, ^2^, ^3^ In addition, it was found that 18%–44% of the affected children's siblings also presented with infraoccluded teeth, which appears to indicate a genetic contribution to this condition.^1^, ^2^, ^3^, ^4^

Infraocclusion can also be categorized as slight, moderate, or severe.^1^, ^2^ The Facile classification, as described by Brearly,^5^ defines infraocclusion as slight when the occlusal surface of the tooth is 1 mm below that of its neighbors, moderate when the occlusal surface of the tooth is level to or below the contact point of the neighboring teeth, and severe when the occlusal surface of the tooth is below the interproximal gingival tissue.^1^ Both the prevalence and severity of infraocclusion increases with chronological age. Therefore, a younger patient who experiences primary tooth ankylosis is likely to demonstrate more severe infraocclusion due to the longer period of vertical alveolar bone height development. Although most ankylosed primary teeth eventually exfoliate with the ongoing eruption of the succedaneous tooth underneath, delayed exfoliation is common. Despite the relative infrequency of unfavorable outcomes, infraoccluded primary molars are known to be associated with other dental anomalies, including hypodontia.^6^ The eruption path of the successor tooth must be monitored closely and periodically to avoid undesirable permanent tooth impactions. In extreme cases, the ankylosed primary tooth can submerge to the point of complete coverage by the surrounding hard and soft tissues as detailed in this case report.^7^

Despite the uncertainty, there is a general consensus that infraocclusion is multifactorial in etiology.^1^, ^2^, ^3^ It has been postulated that infraocclusion may be genetic in nature as this condition can be seen in families; however, there are several factors such as altered local metabolism, local trauma, and infection that may also potentially result in the infraocclusion of teeth.^1^, ^2^, ^3^

Hypodontia, also known as congenitally missing teeth, is another common dental developmental anomaly that indicates that at least one permanent tooth has failed to form and erupt into the oral cavity.^4^, ^8^, ^9^, ^10^, ^11^, ^12^, ^13^, ^14^

The etiology of congenitally missing teeth is also considered to be multifactorial, with genetic and environmental factors potentially both playing a role.^4^, ^8^, ^9^, ^10^, ^11^, ^12^, ^13^, ^14^ Heritability of congenitally missing teeth has been established through familial studies where parents and their offspring and/or siblings have presented with the same or similar congenitally missing teeth.^4^, ^6^, ^8^, ^9^, ^10^, ^11^, ^12^, ^13^, ^14^, ^15^, ^16^, ^17^, ^18^ Numerous developmental syndromes such as Down, Book, and Rieger are associated with congenitally missing teeth, which suggests the influence of genetic factors on the development of the dentition.^6^, ^13^, ^14^, ^15^ It has been suggested that missing anterior teeth have a stronger genetic contribution, while the absence of posterior teeth may be more sporadic in nature.^13^

When reporting on the prevalence of hypodontia, the inclusion or exclusion of the third molars produces a distinct difference in the data.^4^ This difference is due to the high frequency of congenitally missing third molars across all genetic populations.^4^, ^19^ Excluding the third molars, the prevalence of congenitally missing teeth in specific teeth appears to be determined by the population demographics.^4^, ^19^ The prevalence of congenitally missing teeth in the primary dentition has been reported to be <1%; however, the prevalence of missing teeth in the permanent dentition ranges from 1.6% to 36.5%, when the absence of third molars are included. When missing third molars are excluded from the data, the prevalence of congenitally missing permanent teeth is significantly lower and reported to range between 1.6% and 6.9%.^13^ It is also reported that congenitally missing teeth occurs bilaterally in the mouth almost two to three times more frequently than unilaterally. Excluding the third molars, the most common congenitally missing teeth in the permanent dentition are the mandibular second premolars followed by the maxillary lateral incisors.^13^, ^14^

## 2 CASE DESCRIPTION AND DIAGNOSIS

**FIGURE 1:**
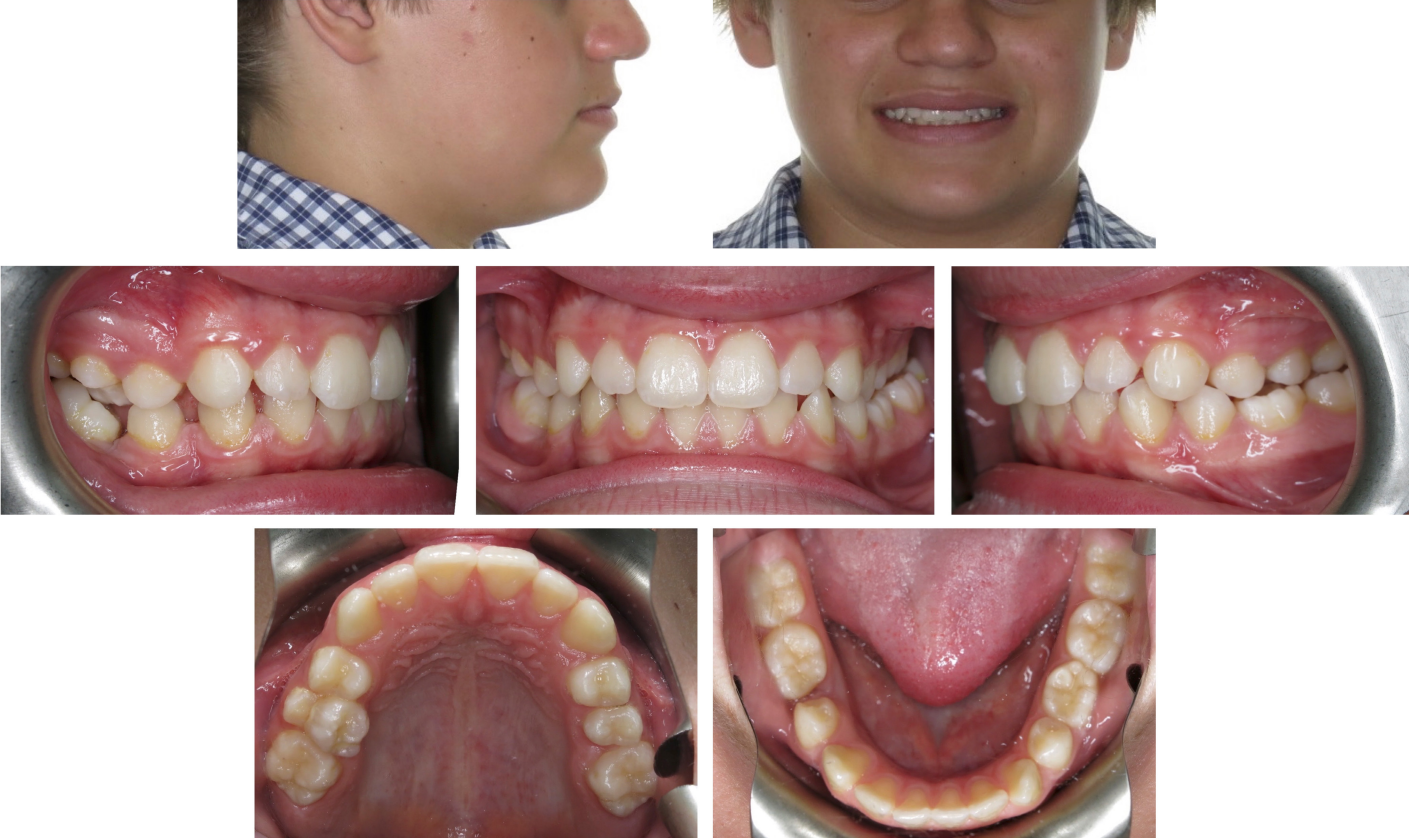
Pre‐treatment photographs of a 14‐year‐old patient demonstrate an atypical Class II indefinite malocclusion with a left‐side posterior lingual crossbite. The 75 is over‐retained and spacing is evident in the fourth quadrant associated with significant mesial crown tipping of the 46.

A 14‐year‐old patient was referred to assess the potential for comprehensive orthodontic treatment. It was noted that the patient was in the late mixed dentition with an atypical Class II indefinite malocclusion. The overjet was determined to be 2 mm and the maxillary incisors covered approximately 25% of the mandibular incisors in the vertical plane. Although the maxillary and mandibular midlines were coincident with each other, both dental midlines were located 2 mm to the right side of the facial midline. The right‐side molar relationship was Class III; however, the right‐side canine relationship was half‐unit Class II. The crown of the mandibular right first permanent molar (46) was also deemed to have a significant mesial tip. The mandibular right second primary molar (85) was not clinically visible, and there was no record of this tooth being extracted. Due to the presence of the mandibular left second primary molar (75), the left‐side molar relationship had a mild Class II tendency, and the left‐side canine relationship was Class I. A posterior lingual crossbite was present on the left‐side and no associated functional shift from the retruded contact position to the position of maximum intercuspation was evident (Figure 1).

**FIGURE 2:**
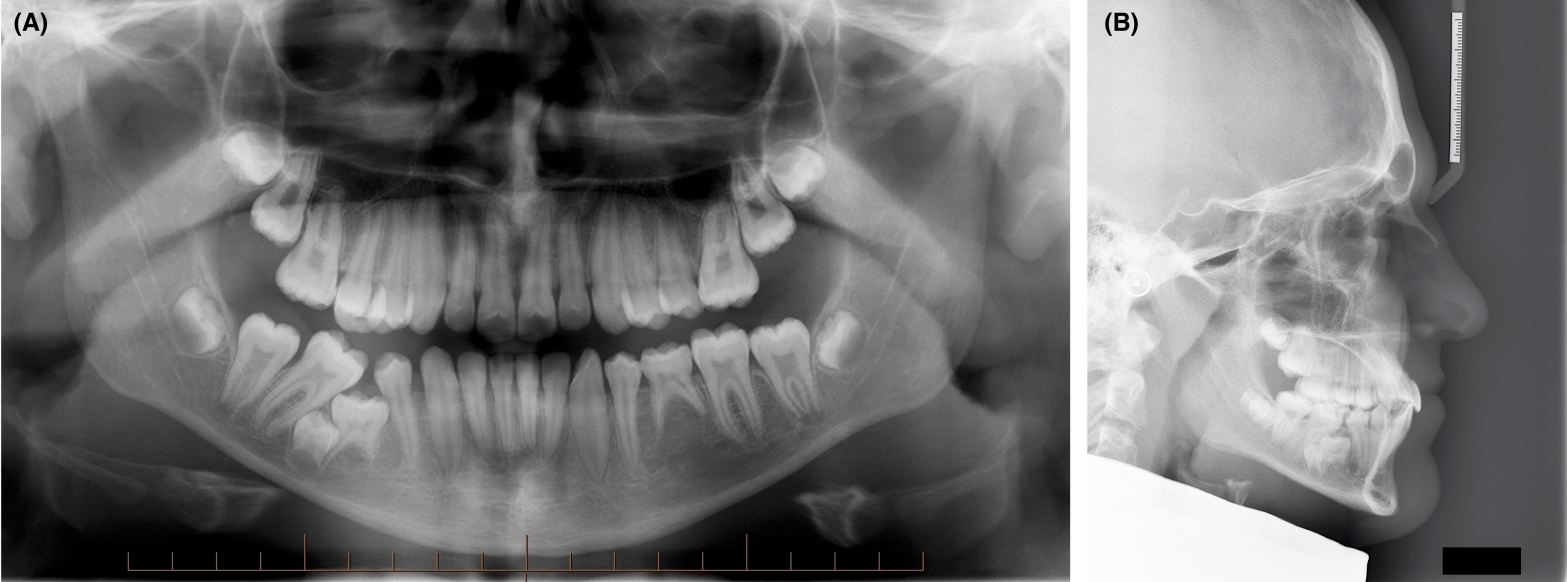
(A) The pre‐treatment panoramic radiograph confirmed that all permanent teeth are present except for the 35. Severe submergence of the 45 is evident and directly associated with impaction of the 45. (B) The pre‐treatment lateral cephalograph revealed a Class I skeletal base relationship and mesofacial vertical facial morphology. The maxillary and mandibular incisor angulations are within the normal range.

A panoramic radiograph (Figure 2A) revealed ankylosis and severe submergence of the 85 along with displacement and impaction of the developing 45. It appeared that the ankylosis and submergence of the 85 had disrupted the alveolar development in this region, which in turn resulted in the localized distortion of the posterior teeth in the fourth quadrant. Despite the highly unusual radiographic appearance in this area, no current or previous pain or discomfort was reported by the patient. It was also noted that the mandibular left second premolar (35) was congenitally missing and the mandibular left second primary molar (75) was over‐retained with significant remaining root structure. The 75 showed no evidence of infraocclusion and was clinically sound and non‐mobile. All third molars were present in the crown stage of development.

A lateral cephalograph and subsequent cephalometric analysis revealed that the patient had a horizontal skeletal Class I relationship, a brachyfacial vertical facial morphology and normal angulation of the maxillary and mandibular incisors. Soft tissue analysis determined that the facial features were symmetrical, along with a pleasing facial profile and competent lip function (Figure 2B).

The relevant diagnostic findings are summarized in Table 1 and as a prioritized problem list in Table 2. Due to the severe submergence of the 85 and impaction of the 45, the patient was referred to an oral and maxillofacial surgeon for assessment of this area and to plan the required surgical management. Several potential treatment options were considered (Table 3). Each of these treatment options have been summarized and compared along with their specific advantages and disadvantages (Table 4).

**TABLE 1:** Key diagnostic features

Skeletal
Class I skeletal base with a mild Class III tendency (Horizontal) Mesofacial growth pattern with a mild brachyfacial tendency (Vertical) Normal facial symmetry (Transverse)
Dental
RHS: ½ unit Class II canine relationship, LHS: Class I molar and canine relationship (Horizontal) Slightly increased overjet ~2 mm (Horizontal) Normal angulation of the maxillary and mandibular incisors Normal anterior overbite ~25% (Vertical) Both maxillary and mandibular dental midlines located 2 mm to the RHS of the facial midline (Transverse) LHS posterior lingual crossbites, indicating a relative maxillary transverse deficiency (Transverse)
Soft tissues
Normal lip position (Horizontal) Normal lip seal (Vertical) Normal chin point position (Transverse)
Other diagnostic features of note
Significant mesial tip crown of the 46 Over‐retained 75 (non‐mobile) Congenitally missing 35 (noted on the panoramic radiograph) 85 was not clinically visible Severely infraoccluded 85 due to historical ankylosis and associated impaction of the 45 (noted on the panoramic radiograph) Mild maxillary arch malalignment Mild mandibular arch spacing in the 4th quadrant Third molar teeth present in the early stage of formation

**TABLE 2:** Systematic problem list

Severely infraoccluded 85 due to historical ankylosis, associated impaction of the 45 and disrupted alveolar development in the fourth quadrant Mandibular arch distortion, arch asymmetry and spacing in the fourth quadrant Over‐retained 75 and congenitally missing 35 LHS posterior lingual crossbite Likely future impaction of the developing maxillary and mandibular third molars

**TABLE 3:** Relevant treatment options

Treatment option 1:
Extract the over‐retained 75, infraoccluded 85, impacted 45 and 18, 28. Comprehensive orthodontic fixed appliance treatment including effective bilateral mandibular molar mesialization. Rapid maxillary expansion (RME) to address the maxillary transverse deficiency and correct the LHS posterior lingual crossbite Removing the 75 will avoid the need for a future implant in this region and effective mesialization of the 36, 37 will create space for the unerupted 38 Removing the 85 and 45 will create an alveolar bony defect which can be regenerated with normal healing and effective mesialization of the 46, 47. Such mesialization will also create space for the unerupted 48 Removing the 18, 28 as these teeth will be either impacted or occlusally redundant post‐treatment Use of TADs can provide safe, predictable and effective orthodontic tooth movements as required (i.e., uprighting of the tipped 46 and effective mandibular molar mesialization) Aims to maintain normal anterior overbite, overjet and the currently harmonious lip positions and incisor angulations Review the unerupted mandibular third molars until their expected eventual clinical eruption
Treatment option 2:
Maintain the over‐retained 75 long‐term. Extract the infraoccluded 85, impacted 45 and 18, 28, 38. Comprehensive orthodontic fixed appliance treatment including effective mandibular molar mesialization on the right side.
Rapid maxillary expansion (RME) to address the maxillary transverse deficiency and correct the LHS posterior lingual crossbite Removing the 85 and 45 will create an alveolar bony defect which can be regenerated with normal healing and TAD‐assisted mesialization of the 46, 47. Effective mesialization will also create space for the unerupted 48 Maintaining the 75 is clinically justifiable as this tooth is structurally sound and demonstrates no evidence of infraocclusion or mobility. Upon its eventual clinical failure, a restorative implant can be placed in this region. This represents simpler orthodontic treatment in the 3rd quadrant Review the unerupted 48 until its expected eventual clinical eruption and the over‐retained 75 for any structural integrity issues
Treatment option 3:
Maintain the over‐retained 75 long‐term. Extract the infraoccluded 85 and the impacted 18, 28, 38, 48. Attempt orthodontic alignment of the impacted 45 in conjunction with comprehensive orthodontic fixed appliance treatment.
Rapid maxillary expansion (RME) to address the maxillary transverse deficiency and correct the LHS posterior lingual crossbite Surgical exposure and subsequent orthodontic traction with a gold chain to disimpact the 45 is likely to be both prolonged and challenging. The 45 may also be significantly damaged during removal of the ankylosed 85 Maintaining the 75 is clinically justifiable as this tooth is structurally sound and demonstrates no evidence of infraocclusion or mobility. Upon its eventual clinical failure, a restorative implant can be placed in this region
Treatment option 4:
Extract the over‐retained 75, infraoccluded 85, impacted 45 and 15, 25 in conjunction with comprehensive orthodontic fixed appliance treatment.
Rapid maxillary expansion (RME) to address the maxillary transverse deficiency and correct the LHS posterior lingual crossbite Removing the 15, 25 can be justified to balance the removal of the 75, 85 Ideally, TAD‐assisted space closure should be performed in both the maxillary and mandibular arches for effective molar mesialization and to avoid any significant incisor retraction (as the current lip position and incisor angulations are deemed to be harmonious and favorable) Review the unerupted maxillary and mandibular third molars until their expected eventual clinical eruption
Treatment option 5:
Extract the infraoccluded 85, impacted 45 and 18, 28. Rather than attempting effective mandibular molar mesialization in the third quadrant, comprehensive orthodontic fixed appliance treatment can be used to facilitate future autotransplantation of the 38 into the congenitally missing 35 region.
Rapid maxillary expansion (RME) to address the maxillary transverse deficiency and correct the LHS posterior lingual crossbite The 38 did not demonstrate sufficient tooth development at the time of the planned removal of the ankylosed 85 and impacted 45 to permit safe autotransplantation under a single general anesthetic Although future third molar autotransplantation would significantly reduce the orthodontic tooth movement required in the third quadrant, the autotransplantation procedure must be delayed until an ideal stage of third molar root development is present (at approximately 18 years of age) Although contemporary tooth autotransplantation techniques have a high success rate, post‐transplant complications are still possible

**TABLE 4:** Comparison of the relevant treatment options

	Treatment option 1	Treatment option 2	Treatment option 3	Treatment option 4	Treatment option 5
		Maintain 75	Maintain 75		
	Extract 75, 85, 45 and 18, 28	Extract 85, 45 and 18, 28, 38	Extract 85 and 18, 28, 38, 48	Extract 75, 85, 15, 25, 45	Extract 85, 45 and 18, 28
	Bilateral TADs	Unilateral TAD	Attempt disimpaction of the 45	Bilateral TADs	Unilateral TAD future autotransplantation of 38
Maintain lip and incisor positions	Yes	Yes	Yes	Yes	Yes
Avoids the need for future restorative implants	Yes	No	No	Yes	Yes
Regenerates alveolar bone in quadrant 4	Yes	Yes	Yes	Yes	Yes
Facilitate future third molar eruption	Yes (38, 48)	Yes (48)	No	Yes (18, 28, 38, 48)	Yes (48)
Orthodontic treatment difficulty	++	+	+++	+++	+

Following extensive communication between the orthodontist, general dentist, and the oral and maxillofacial surgeon, it was determined that the most predictable treatment option involved removal of the submerged 85 and the impacted 45. The oral and maxillofacial surgeon indicated that removal of the ankylosed 85 would necessitate significant bone removal and the impacted 45 would be likely to be damaged during this process. In addition, surgical exposure and subsequent orthodontic traction with a gold chain to disimpact the 45 was expected to be both prolonged and challenging. As the 35 was also congenitally missing, a decision was also required regarding the over‐retained 75. Given that the mandibular third molars were developing and that over‐retained primary teeth generally have reduced longevity compared with healthy permanent teeth, removal of the 75, uprighting of the 46 and complete orthodontic space closure through bilateral mandibular molar mesialization was recommended. This treatment option would require the use of temporary anchorage devices (TADs) to ensure maximum predictability of these challenging orthodontic tooth movements. Complete orthodontic space closure in the mandibular arch through bilateral mandibular molar mesialization would be associated with the numerous potential benefits for the patient. Firstly, this option would eliminate the need for future prosthodontic replacement for the congenitally missing 35 and the irretrievable 45. The process of orthodontic tooth movement has been shown to be osteo‐inductive and has the ability to regenerate atrophic alveolar bone resulting from congenital absence of teeth and/or iatrogenic issues. Effective mandibular molar mesialization would also protect the harmonious pre‐treatment incisor angulation and lip positions whilst also creating significant space for spontaneous future eruption of the developing third molar teeth into functional positions.

## 3 ORTHODONTIC TREATMENT PROGRESS

The patient exhibited satisfactory oral hygiene and was motivated to undergo orthodontic treatment. General dental examinations and preventative treatment were provided at six‐month intervals during the comprehensive orthodontic treatment.

### 3.1 Placement of fixed appliances

Comprehensive orthodontic treatment commenced with a placement of a Hyrax rapid maxillary expansion appliance to correct the left‐side posterior lingual crossbite and pre‐adjusted maxillary labial fixed appliances (0.022″ × 0.028″ slot, MBT prescription, 3M Oral Care). Instructions were given to turn the rapid maxillary expansion appliance once daily for 26 consecutive days. Following another review, the maxillary arch expansion was deemed to be satisfactory.

### 3.2 Four months

The rapid maxillary expansion appliance was removed, and bonded molar tubes were placed on the 16 and 26. The patient had a general anesthetic 3 weeks later, where the oral and maxillofacial surgeon removed the 75, 85, 18, 28, and 45. Temporary anchorage devices (1.8 × 8.0 mm – Synthes® Solothurn) were placed between the mandibular canines and first premolars to facilitate complete orthodontic space closure in the mandibular arch.

### 3.3 Nine months

**FIGURE 3:**
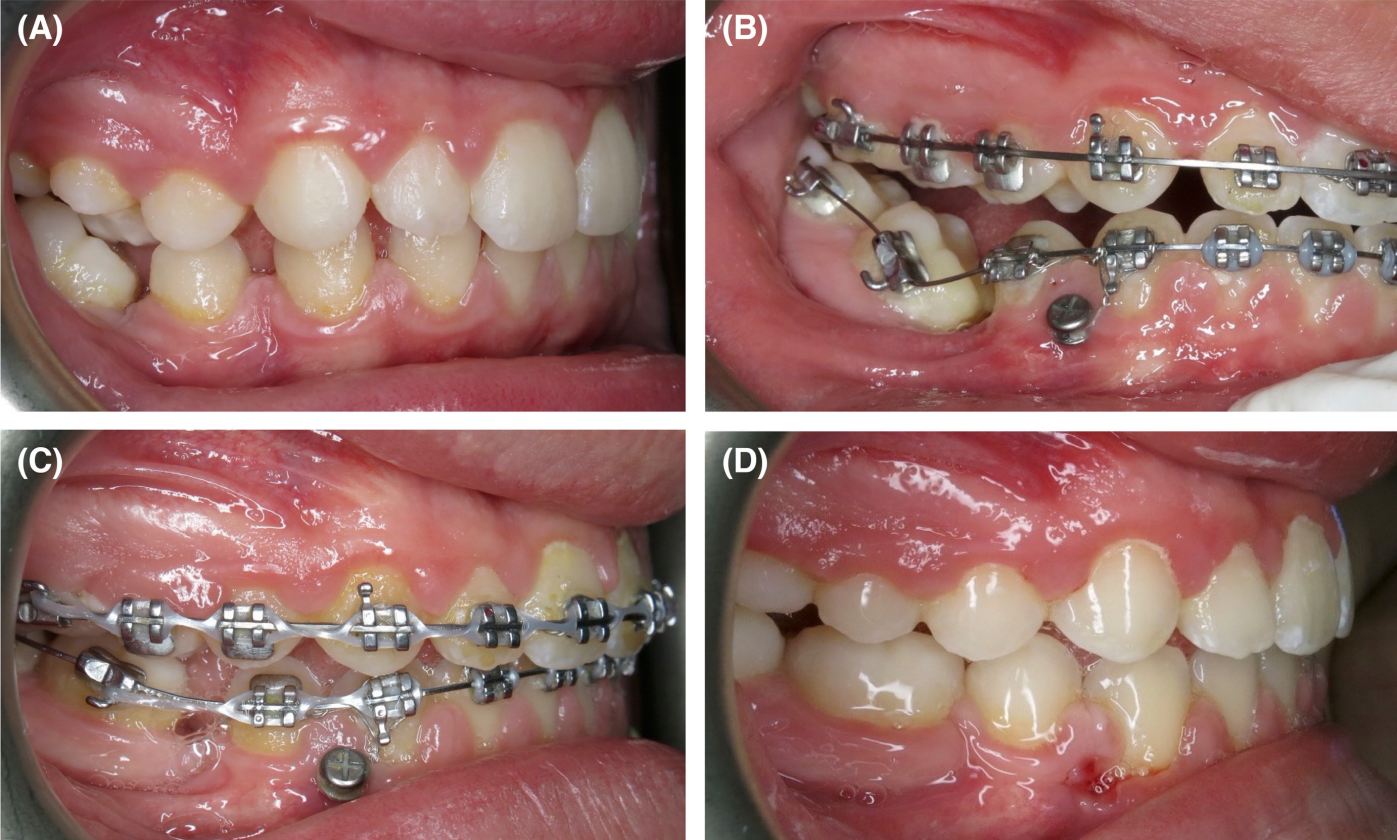
(A) The pre‐treatment right‐side photograph demonstrates significant mesial tip of the 46 crown and interdental spacing in the fourth quadrant; (B) Progress photograph taken at 9 months demonstrates the hybrid mandibular orthodontic archwire set‐up to stabilize the 43 and 44 whilst uprighting the 46; (C) Progress photograph taken at 15 months reveals effective uprighting and mesial movement of the 46 and 47; (D) Completion of active orthodontic treatment at 28 months.

Due to the significant mesial crown tip of the 46, a hybrid mandibular archwire technique was implemented (Figure 3). The rigid rectangular (0.016″ × 0.022″) stainless steel archwire ended at the distal aspect of the 44. A sectional flexible nickel‐titanium (0.016″) wire was placed from the 43 to the 46 and was located underneath the rectangular steel archwire in the 43 and 44 regions. The TAD between the 43 and 44 was connected to the 43 and 44 brackets with a fine stainless steel ligature wire. This ligature wire, along with the rectangular stainless steel archwire, provided stabilizing anchorage for the 43 and 44, as the reciprocal biomechanical force generated from the uprighting of the mesially tipped 46 was expected to be significant. These fixed appliance components provided effective stabilization to prevent undesirable movement of the 43 and 44.

### 3.4 15 months

**FIGURE 4:**
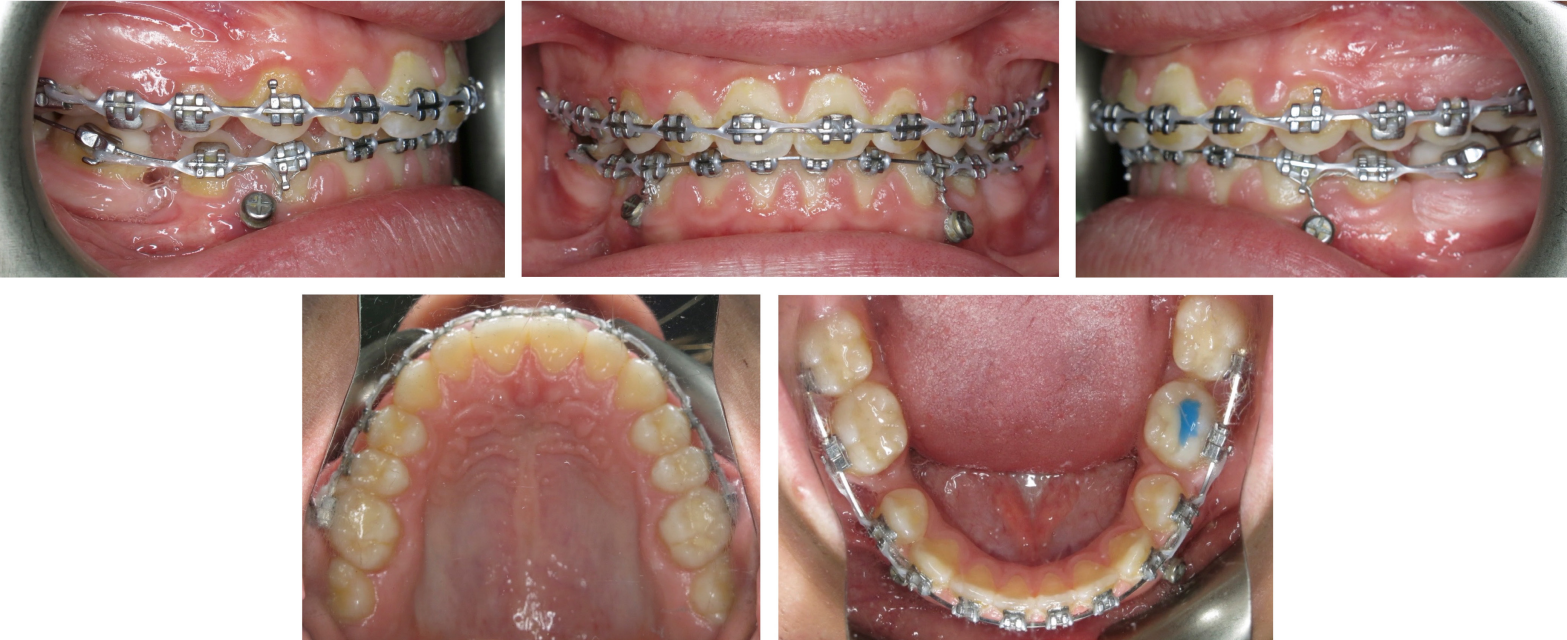
Progress intra‐oral photographs obtained 15 months into active treatment. Note the effective mesialization of the mandibular molar teeth, along with bone and soft tissue regeneration in the 45 extraction site.

**FIGURE 5:**
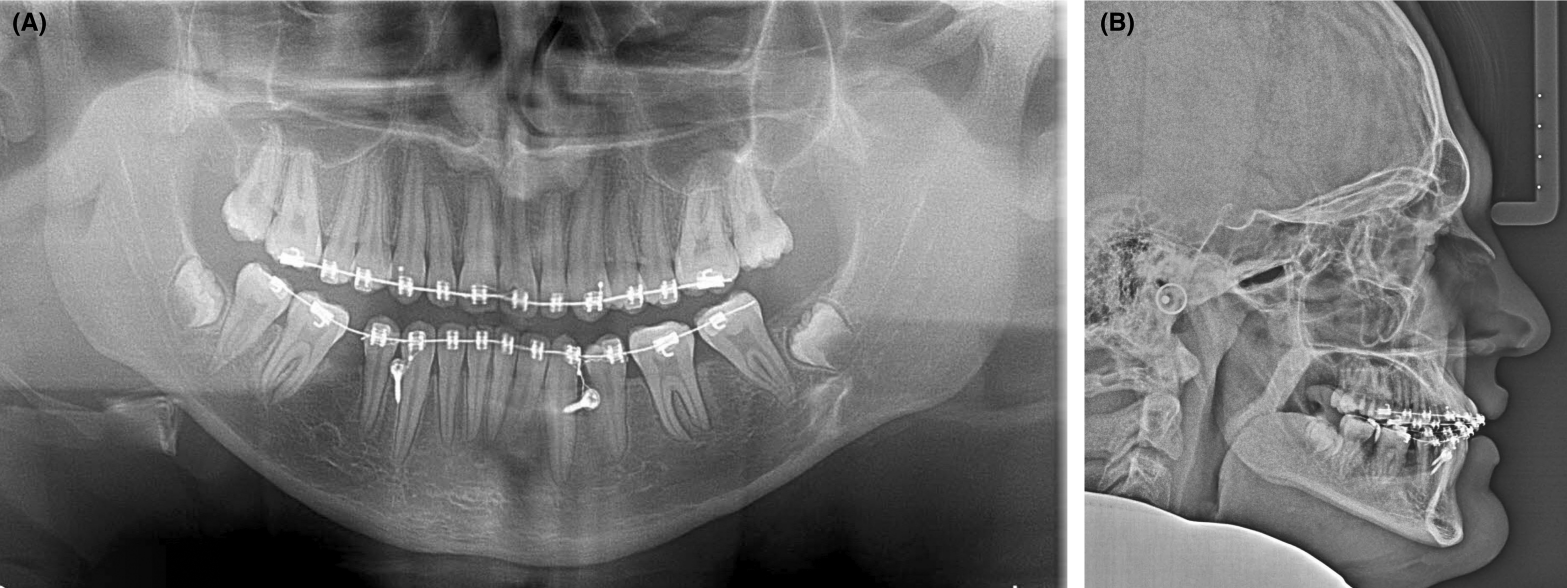
(A) Progress panoramic radiograph obtained 15 months into active treatment. Effective mesialization of the mandibular first and second molars is evident, along with satisfactory overall root parallelism; (B) The progress lateral cephalograph obtained 15 months revealed maintenance of the maxillary and mandibular incisor angulations.

The 46 was successfully uprighted and mesial movement of the mandibular molar teeth progressed without incident (Figures 4 and 5). The ligature wire remained in place bilaterally to facilitate the remaining mesial movement without undue distal movement of the mandibular anterior teeth. The alveolar bone and soft tissue regeneration associated with the mesial movement of the 46 was deemed to be very favorable.

### 3.5 28 months

**FIGURE 6:**
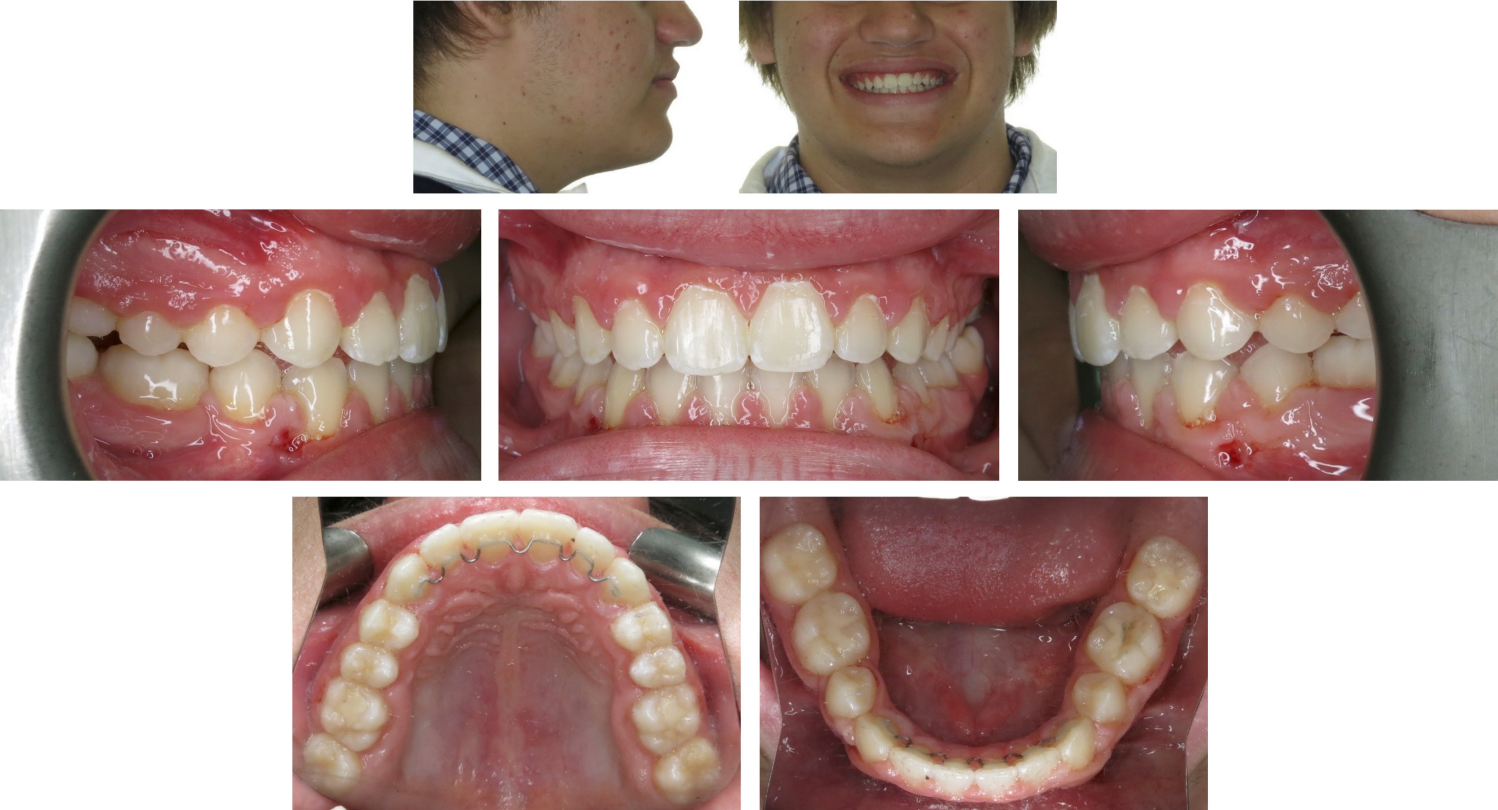
Photographs obtained at the completion of 28 months of active orthodontic treatment. The temporary anchorage devices (TADs) in the third and fourth quadrants were removed on the same day.

The fixed labial orthodontic appliances were removed and maxillary and mandibular flossable fixed retainers were bonded on the anterior teeth to reduce the potential for rotational relapse (Figure 6). Maxillary and mandibular vacuum‐formed Essix® C+ material (Dentsply Raintree Essix®) removable retainers were also issued for indefinite nocturnal wear.

### 3.6 53 months (25 months post‐deband)

**FIGURE 7:**
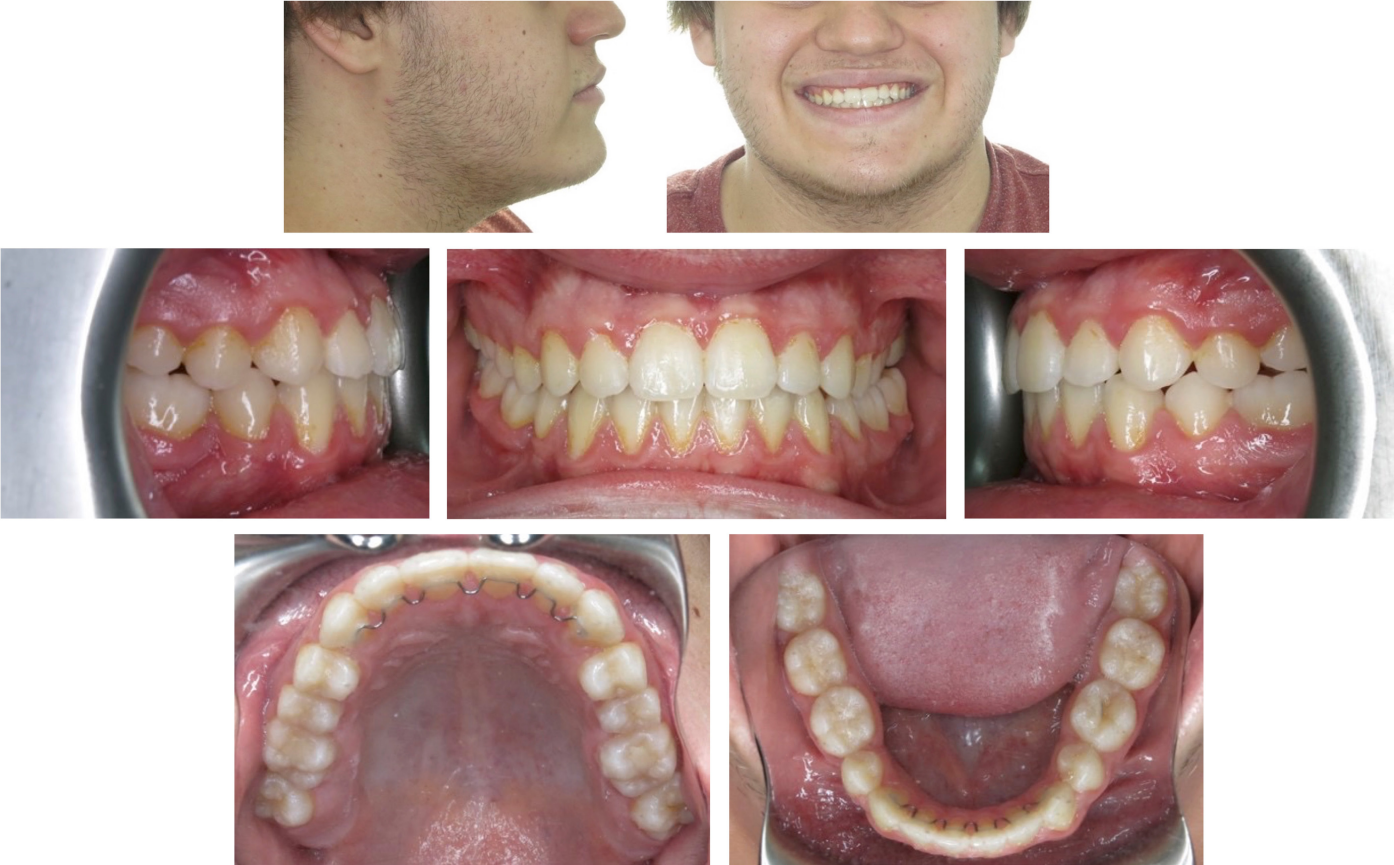
Photographic records obtained 25 months after the completion of active treatment. The patient was 20 years old at this time. Note the spontaneous eruption of the 38 and 48.

**FIGURE 8:**
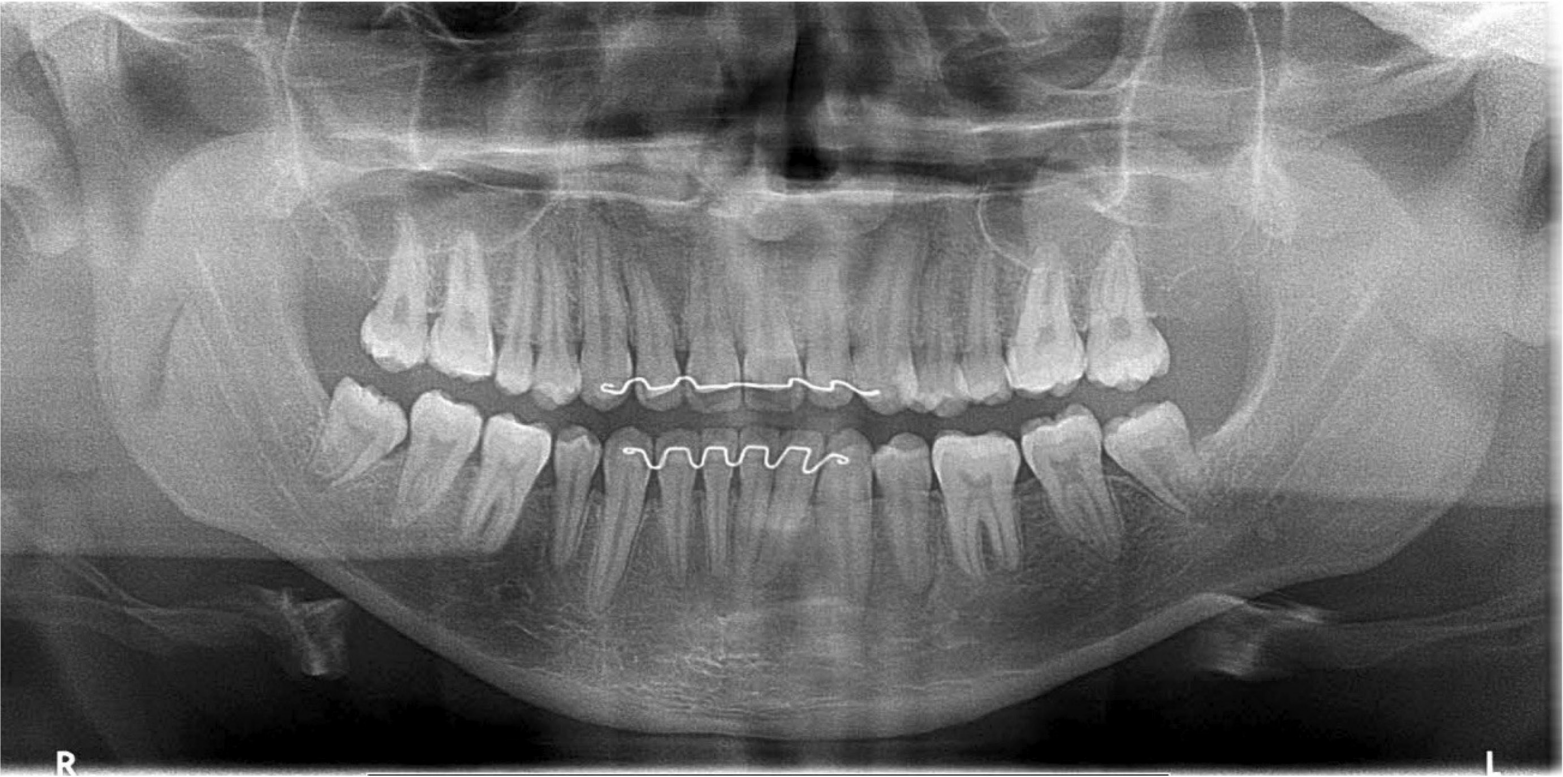
Panoramic radiograph obtained 25 months after the completion of active treatment. Note the alveolar bone regeneration in the fourth quadrant and improved angulation and vertical position of the 38 and 48.

The patient was reviewed 25 months following the completion of active orthodontic treatment. (Figure 7). The overall alignment and tooth interdigitation had been maintained. The 26 was noted to be in a posterior lingual crossbite position; however, this minor irregularity did not present any aesthetic or functional concerns. The 38 and 48 were noted to be partially erupted with a very high likelihood of gaining acceptable antagonistic contact with the opposing 17 and 27. An updated panoramic radiograph (Figure 8) demonstrated that alveolar bone regeneration had occurred in the fourth quadrant and that the eruption path of the developing 38 and 48 appeared favorable.

## 4 DISCUSSION

This case report has detailed a pleasing overall treatment outcome which addressed the presenting malocclusion and eliminated the future need for prosthodontic treatment for the congenitally missing 35 and 45. The spontaneous eruption of the 38 and 48 into satisfactory clinical positions further enhanced the treatment outcome from a functional perspective. The eruption of the 38 and 48 was made possible through effective mesialization of the mandibular first and second permanent molars during the orthodontic treatment. Such mesial movement created space distal to the mandibular second molars which subsequently created an unobstructed eruption path for the 38 and 48. Although an argument could be made for improving the alignment of the 38 and 48 with a second phase of fixed appliances, this was not desired by the patient. Should the 38 and/or 48 not erupt further and require removal for any dental health reason, such removal would be undoubtedly more straightforward compared with their original pre‐treatment position and angulation (Figure 2B). This improvement in angulation and vertical position can be attributed to the effective orthodontic mesialization of the mandibular first and second permanent molars.

The mandibular right second primary molar tooth was not visible clinically at the initial orthodontic consultation. The pre‐treatment panoramic radiograph was rather confronting, as it revealed a severe submergence of the 85 and its resultant effect upon the succedaneous 45. This problematic outcome emphasizes the need for routine and periodic assessment of growing patients. The dentition of a growing patient is as an individual and dynamic entity which requires all dental practitioners to be familiar with the eruption sequence of teeth. Any deviation from the norm should be diagnosed and managed in a timely manner, in order to preserve dental arch integrity and to avoid negative outcomes.^20^ In retrospect, earlier diagnosis of the infraoccluded 85 (i.e., when this tooth was still in a supragingival position) would have been ideal and may have completely avoided the surgical and orthodontic management challenges encountered in the fourth quadrant.

Congenitally missing premolar teeth are relatively common and are often associated with both short and long‐term clinical dilemmas. Numerous factors must be carefully considered in the diagnostic and treatment planning process. It is imperative that every relevant treatment option be evaluated from the cost–benefit perspective and thoroughly discussed with the patient as part of the informed consent process.

Maintaining an over‐retained primary molar when the succedanenous premolar is congenitally missing may be a reasonable option for some individual patients, particularly if the primary molar demonstrates no sign of infraocclusion, is unrestored, non‐mobile and has significant remaining root structure. Such a tooth may be expected to have a good long‐term prognosis and decent clinical longevity. It has been reported that after the age of 20 years, retained primary molars display minimal change with respect to infraocclusion, tipping of adjacent teeth and root resorption. Therefore, should a primary molar still be present in the dental arch at 20 years of age, it may have a good prognosis for long‐term survival.^21^

However, it is difficult to argue that an intact primary tooth would have superior clinical longevity in comparison with a healthy permanent tooth. For some patients, it may be advantageous to remove primary molars and appropriately close the extraction spaces in conjunction with orthodontic treatment. Such space redistribution may create sufficient space for third permanent molars to successfully erupt, as demonstrated in this case report.

Anchorage is defined as a resistance to unwanted tooth movement^22^ and anchorage control is a fundamental principle of contemporary orthodontic treatment.^7^, ^23^ Anchorage can be controlled through the use of intra‐oral or extra‐oral devices. The development of non‐osseointegrating titanium bone screws has revolutionized orthodontic anchorage control as they offer a simple, safe, and effective option with reduced patient compliance.^24^ These mechanically retained bone screws are generically referred to as temporary anchorage devices (TADs) and due to their placement into inter‐radicular, mandibular, or palatal bone, their clinical application is also known as skeletal anchorage.^7^, ^22^, ^23^, ^24^

The use of TADs can permit safe, predictable, and effective orthodontic mesialization of the mandibular first and second permanent molars. Although such movement can also be achieved through the implementation of intermaxillary elastics or bite correcting springs, TADs can significantly reduce the amount of patient compliance required for challenging tooth movements. With the benefit of hindsight, the authors concede that the TADs used for this patient may have provided better indirect anchorage control if rigid wire splinting was used between the TADs and the mandibular canines or premolar teeth.^25^, ^26^ In addition, mesial force applied directly to the TADs could also have been implemented following uprighting of the 46.^27^

The mesialization of first and second permanent molars to provide space for third molars has been widely discussed in the literature. When a premolar or a second primary molar is extracted and the remaining posterior teeth have been moved mesially, the probability of spontaneous eruption of the third molars does increase.^28^, ^29^, ^30^, ^31^, ^32^, ^33^ In general, the greater the distance of first and second molar mesialization, the greater the probability of spontaneous normal eruption of the third molar teeth.^28^

Removal of significant alveolar bone was required to extract the ankylosed 85 and the impacted 45. Fortunately, subsequent spontaneous bone regeneration generally allows bony defects to fill with bone without the presence of a stimulus or grafting material.^34^ This phenomenon also occurs in patients who have had large cysts removed from the ramus of the mandible. In addition, orthodontic tooth movement through the area of an alveolar bony defect can further facilitate regeneration of bone.^34^ Orthodontic tooth movement can both stimulate and help maintain bone formation; however, how this process occurs is not completely understood.^35^ Two hypotheses exist with respect to the process of osteogenesis. The first hypothesis is a bone bending theory as described by Epker and Frost,^36^, ^37^ which postulates that the flexion of the alveolar bone creates convexities and concavities which in turn stimulates osteogenesis and resorption. The second hypothesis is known as the pressure‐tension hypothesis.^38^, ^39^ This hypothesis relates to the concept of creates pressure and tension areas that occur within the periodontal ligament as the tooth moves. The pressure and tension stimulate simultaneous bone resorption and osteogenesis which then allows for tooth movement. For the patient described in this case report, the large bony defect which resulted from surgical removal of the 85 and 45 was completely resolved, and likely occurred through a combination of normal bone healing and by the osteogenic effects of orthodontic tooth movement.

In recent times, there has been a resurgence in the technique of tooth autotransplantation, which refers to the technique of transplanting embedded, impacted or erupted teeth from one site into another in the same individual.^40^ Autotransplantation can provide a cost‐effective and biocompatible solution for adolescent patients with congenitally missing teeth or significantly compromised teeth when a suitable donor tooth is available.^41^ Although carefully considered individual case selection and surgical skill are the critical determinants for success, advances in three‐dimensional computed tomography and rapid prototyping have the potential to significantly reduce the technique sensitivity of the autotransplantation procedure.^41^

The patient in this case report was also considered to be a potential candidate for tooth autotransplantation. The left mandibular third molar (38) may have eventually become a reasonable donor tooth to replace the congenitally missing 35. However, the 38 did not demonstrate sufficient tooth development at the time of the planned removal of the ankylosed 85 and impacted 45 to permit safe autotransplantation under a single general anesthetic. The clinicians involved in the management of this case determined that effective mesialization of the mandibular molars would result in a very high likelihood of successful eruption of the 38. The favorable third molar eruption outcome which was attained with the orthodontic treatment ultimately justified the clinical decisions made at the treatment planning stage.

Although restorative implants are increasing in acceptance and popularity amongst both patients and clinicians, such treatment may not always represent the most ideal option for compromised or congenitally missing teeth. Actively growing adolescent patients may also pose several management issues for future implant placement.^42^ Given this patient's relatively young age, presence of third molars of suitable morphology and the need for comprehensive orthodontic treatment, maintaining the over‐retained 75 with a view to eventual implant replacement was not considered to be the ideal option for this individual patient.

## 5 CONCLUSION

This case report documents the management of a severely infraoccluded primary molar, an impacted second premolar and a congenitally missing premolar tooth which resulted in significant surgical and orthodontic challenges. Comprehensive planning and careful orthodontic treatment, including use of temporary anchorage devices (TADs), effectively addressed the presenting malocclusion. Although this ambitious orthodontic treatment did not attain an ideal occlusion, favorable outcomes were achieved with respect to alveolar bone regeneration, avoidance of future prosthodontic rehabilitation and spontaneous mandibular third molar eruption.

## AUTHOR CONTRIBUTIONS

AN and PG were the authors and collaborators. DO was the clinician, author and involved in data collection and analysis.

## CONSENT

Written informed consent was obtained from the patient to publish this report in accordance with the journal's patient consent policy.

## Data Availability

Not applicable.
